# Supplementary material for: Anti-CSF-1R therapy with combined immuno-chemotherapy coordinate an adaptive immune response to eliminate macrophage enriched triple negative breast cancers
Source: Nat Commun. 2026 Jan 3;17:1193. doi: 10.1038/s41467-025-67964-2 (PMC12858953; doi:10.1038/s41467-025-67964-2)
Supplement: Supplementary file 1 — Supplementary Information [file 41467_2025_67964_MOESM1_ESM.pdf]

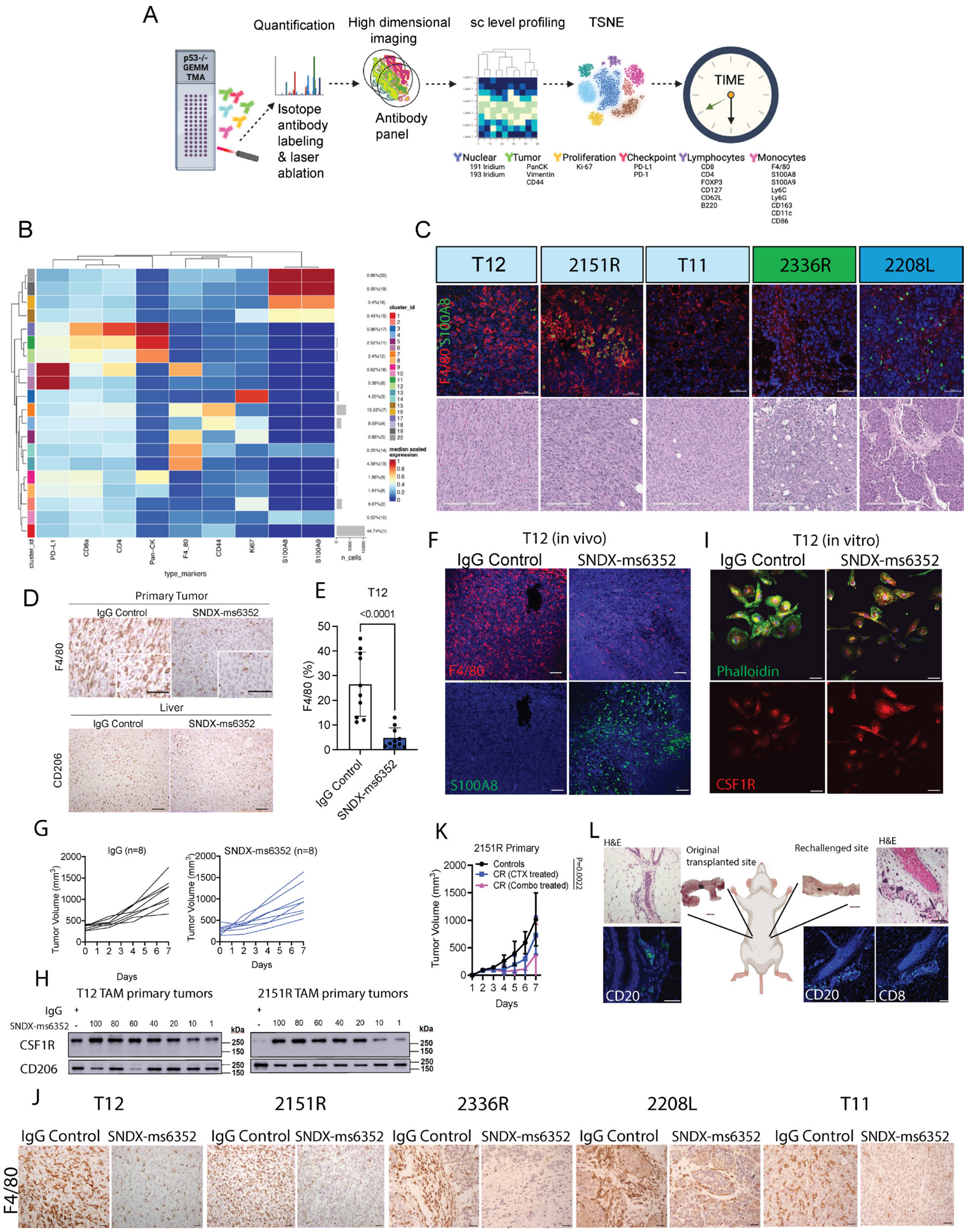
A
Quantification
High dimensional imaging
sc level profiling
TSNE
p53-/- GEMM TMA
Isotope antibody labeling & laser ablation
Antibody panel
TIME
Nuclear
191 Iridium
193 Iridium
Tumor
PanCK
Vimentin
CD44
Proliferation
Ki-67
Checkpoint
PD-L1
PD-1
Lymphocytes
CD8
CD4
FOXP3
CD127
CD62L
B220
Monocytes
F4/80
S100A8
S100A9
Ly6C
Ly6G
CD163
CD11c
CD86
B
cluster_id
median scaled expression
type_markers
n_cells
PD-L1
CD8a
CD4
Pan-CK
F4_80
CD44
Ki67
S100A8
S100A9
C
T12
2151R
T11
2336R
2208L
F4/80 S100A8
D
Primary Tumor
IgG Control
SNDX-ms6352
F4/80
Liver
CD206
E
T12
<0.0001
F4/80 (%)
IgG Control
SNDX-ms6352
F
T12 (in vivo)
IgG Control
SNDX-ms6352
F4/80
S100A8
I
T12 (in vitro)
Phalloidin
CSF1R
G
IgG (n=8)
SNDX-ms6352 (n=8)
Tumor Volume (mm3)
Days
H
T12 TAM primary tumors
2151R TAM primary tumors
IgG
SNDX-ms6352
100 80 60 40 20 10 1
kDa
CSF1R
CD206
250
150
K
2151R Primary
Controls
CR (CTX treated)
CR (Combo treated)
P=0.0022
Tumor Volume (mm3)
Days
L
H&E
Original transplanted site
Rechallenged site
CD20
CD8
J
T12
2151R
2336R
2208L
T11
IgG Control
SNDX-ms6352
F4/80

Supplementary Figure 1. CSF-1R inhibition coupled with CTX leads to a durable response in primary macrophage enriched TNBC pre-clinical models. (A) Schematic illustration of IMC protocol, including isotope labeling, laser ablation and the antibody body panel that includes marker that identify tumor, proliferation, checkpoint, lymphocytes and monocytes. Analysis including single cell level profiling and TSNE were utilized to study the TIME. (B) Heatmap depicting 20 different clusters (cluster_id) from individual protein markers with the respective number of cells (n_cells) detected. (C) Double IF staining of macrophages (F4/80) and neutrophils (S100A8) from claudin-low (T12, 2151R and T11), basal-like (2336R) and luminal-like (2208L) syngeneic model. (D) IHC staining of macrophages (F4/80) in tumor and tissue resident macrophages (CD206) in liver tissues following IgG control or SNDX-ms6352 treatment in T12 model. (E) IHC quantification of macrophages (F4/80) following IgG control or SNDX-ms6352 treatment in T12 model. Statistical analysis performed using 2-tailed, unpaired Student's t test. (n = 8). (F) IF staining of macrophages (F4/80) and apoptosis (Cleaved Caspase 3) following IgG control or SNDX-ms6352 treatment in T12 model ($n = 3$ mice per treatment). (G) Tumor volume following IgG control or SNDX-ms6352 treatment in T12 model. ($n = 8$ mice per treatment). (H) Immunoblots of TAMs isolated from T12 and 2151R TAMs treated with increasing dose of IgG control or SNDX-ms6352. (I) IF staining of cytoskeleton (phalloidin) and CSF-1R marker following IgG control or SNDX-ms6352 treatment in isolated TAMs *in vitro* ($n = 3$). (J) IHC staining of macrophages (F4/80) following IgG Control of SNDX-ms6352 treatments across several Claudin-low (T12, 2151R, T11), basal-like (2336R) and luminal-like (2208L) models ($n = 3$ mice per treatment). (K) Tumor volume of 2151R contralateral tumor cell rechallenged mice post CTX or combination treatment. Statistical analysis performed using Two-way ANOVA, (n = 2-5 mice per treatment). (L) H&E and IF staining of B cell (CD20) and T cell (CD8) following contralateral tumor cell rechallenged mice post combination treatment in the T12 model (n = 3 mice). Data shown as mean ±SEM.

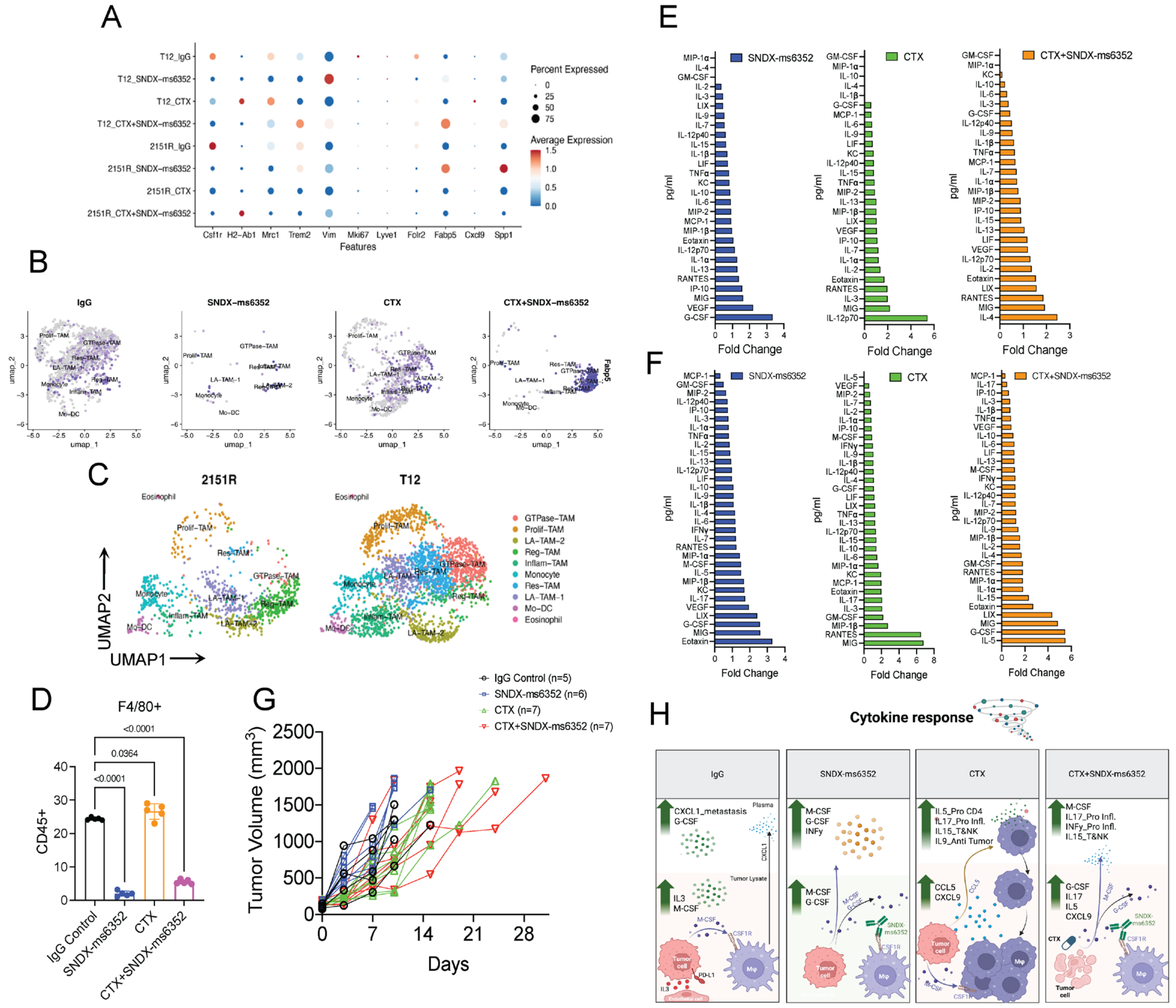

A
B
C
D
E
F
G
H
Percent Expressed
Average Expression
Features
IgG
SNDX-ms6352
CTX
CTX+SNDX-ms6352
2151R
T12
UMAP2
UMAP1
F4/80+
CD45+
IgG Control
Tumor Volume (mm3)
Days
IgG Control (n=5)
SNDX-ms6352 (n=6)
CTX (n=7)
CTX+SNDX-ms6352 (n=7)
pg/ml
Fold Change
Cytokine response

Supplementary Figure 2. CSF-1R inhibition coupled with CTX leads to a durable response in primary macrophage enriched TNBC pre-clinical models. (A) scRNA-seq transcriptomic expression of TAM markers from T12 and 2151R models following IgG control, SNDX-ms6352, CTX and the combination. (B) UMAP clustering from scRNA-seq of *Fabp5* expression levels in various residual TAMs following IgG control, SNDX-ms6352, CTX and the combination in the T12 model. (C) UMAP clustering of different TAM populations identified via scRNA-seq within the 2151R and T12 models. (D) Flow cytometry of F4/80^{+} macrophages within CD45^{+} immune cells in the T12 model following IgG control, SNDX-ms6352, CTX and the combination. Statistical analysis performed using Two-way ANOVA, (n = 5 mice per treatment). (E) Cytokine array profiling from the plasma of SNDX-ms6352, CTX and combination treated mice. Median expression levels compared to IgG controls (fold change), (n = 3 mice per treatment). (F) Cytokine array profiling from the tumor lysate of T12 IgG control, SNDX-ms6352, CTX and combination treated mice. Median expression levels compared to IgG controls (fold change) (n = 3 mice per treatment). (G) Tumor volume of T12 tumors transplanted into immunodeficient athymic nude mice and treated with IgG control, SNDX-ms6352, CTX or combination. (n = 5-7 mice per treatment). (H) Illustration depicting cytokine response observed in plasma and tumor homogenates following each treatment in mice bearing T12 primary tumors. Data shown as mean ±SEM.

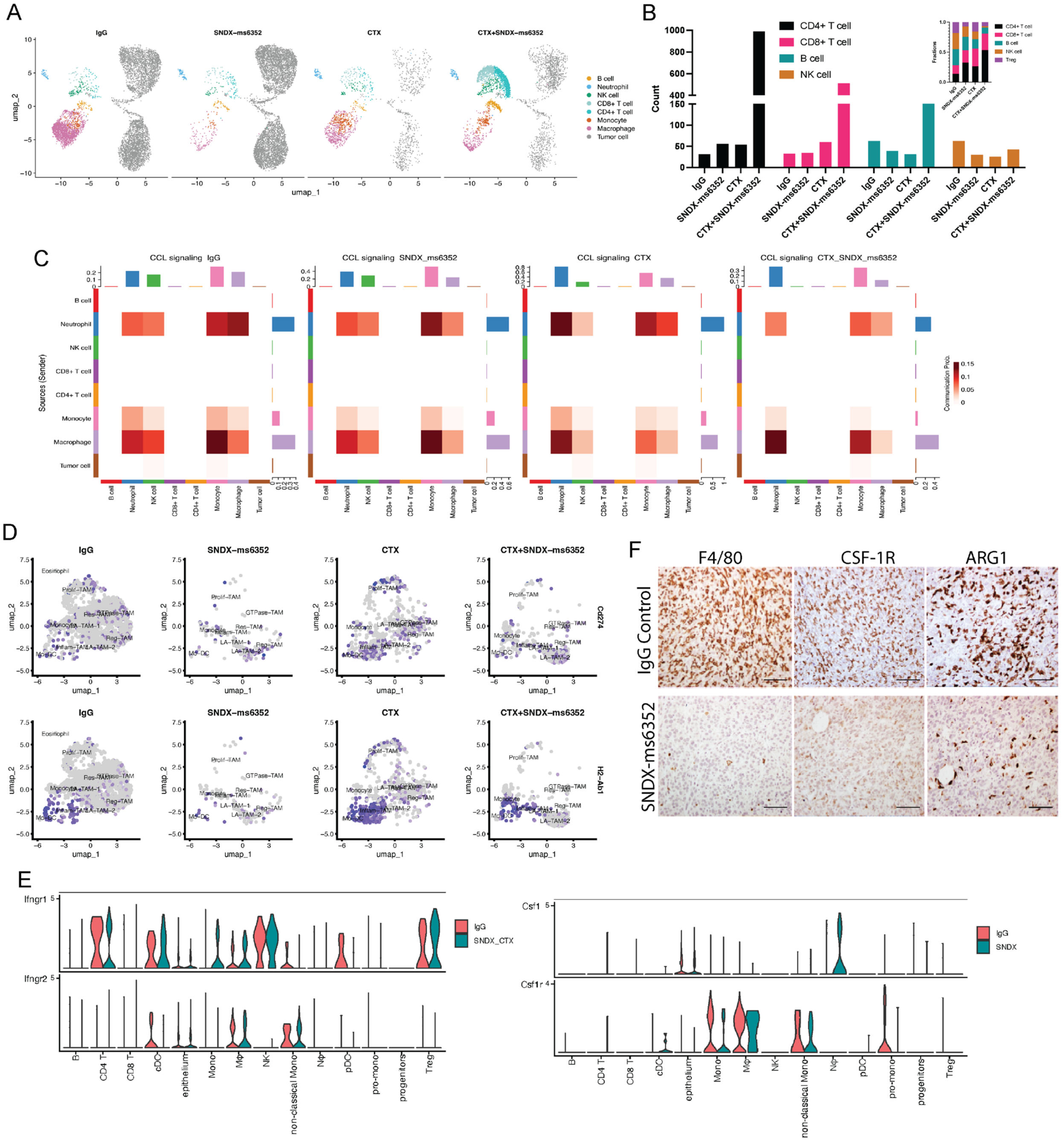

A
IgG
SNDX–ms6352
CTX
CTX+SNDX–ms6352
umap_1
umap_2
B cell
Neutrophil
NK cell
CD8+ T cell
CD4+ T cell
Monocyte
Macrophage
Tumor cell
B
CD4+ T cell
CD8+ T cell
B cell
NK cell
Count
IgG
SNDX-ms6352
CTX
CTX+SNDX-ms6352
Fractions
Treg
C
CCL signaling IgG
CCL signaling SNDX_ms6352
CCL signaling CTX
CCL signaling CTX_SNDX_ms6352
Sources (Sender)
Communication Prob.
D
Eosinophil
Prolif–TAM
GTPase–TAM
Res–TAM
Monocyte
LA–TAM–1
LA–TAM–2
Reg–TAM
Inflam–TAM
Mo–DC
Cd274
H2–Ab1
E
Ifngr1
Ifngr2
IgG
SNDX_CTX
B
CD4 T
CD8 T
cDC
epithelium
Mono
Mφ
NK
non-classical Mono
Nφ
pDC
pro-mono
progenitors
Treg
Csf1
Csf1r
SNDX
F
F4/80
CSF-1R
ARG1
IgG Control
SNDX-ms6352

Supplementary Figure 3. CSF-1R inhibition coupled with CTX leads to a durable response in primary macrophage enriched TNBC pre-clinical models. (A) UMAP visualization of specific cell clusters including CD4, CD8, NK and B cells, post 10-day (short-term) treatment of IgG Control, CTX, SNDX-ms6352 or CTX+SNDX-ms6352 in T12 and 2151R primary tumors (*n* = 3 independently treated mouse tumors). (B) Cellular counts and total fractions of CD4, CD8, B and NK cells following IgG Control, SNDX-ms6352, CTX or CTX+SNDX-ms6352. (C) Heatmaps depicting CCL cell-cell communication signaling following IgG Control, SNDX-ms6352, CTX and CTX+SNDX-ms6352. (D) UMAP clustering from scRNA-seq of *CD274 and H2-Ab1* expression levels in various residual TAMs following IgG control, SNDX-ms6352, CTX and the combination in the T12 model. (E) Bar graph depicting *Ifngr1/2* following IgG Control and CTX+SNDX-ms6352 and *Csf1/Csf1r* following IgG Control and SNDX-ms6352.

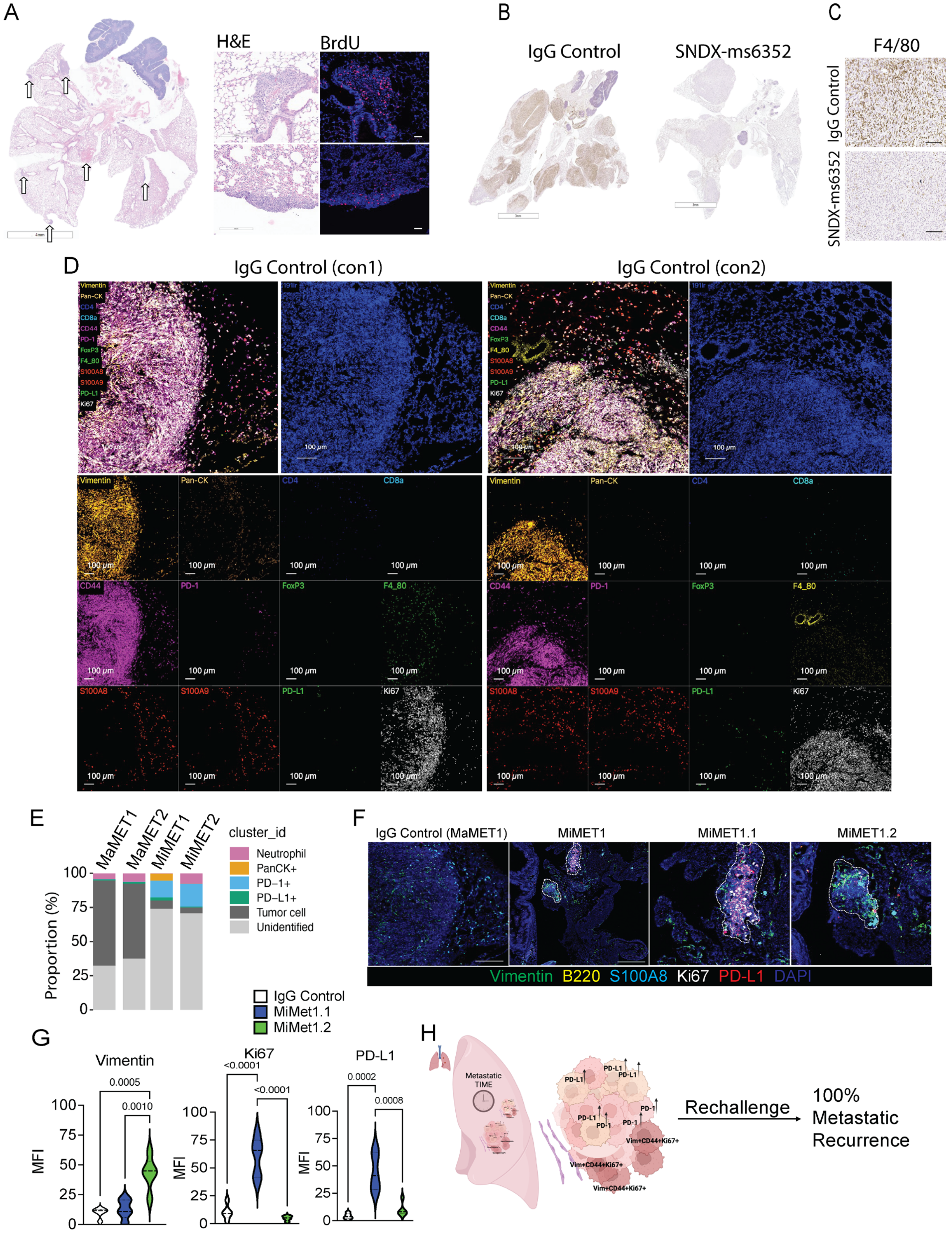

A
H&E
BrdU
B
IgG Control
SNDX-ms6352
C
F4/80
IgG Control
SNDX-ms6352
D
IgG Control (con1)
IgG Control (con2)
Vimentin
Pan-CK
CD4
CD8a
CD44
PD-1
FoxP3
F4_80
S100A8
S100A9
PD-L1
Ki67
100 μm
E
MaMET1
MaMET2
MiMET1
MiMET2
cluster_id
Neutrophil
PanCK+
PD−1+
PD−L1+
Tumor cell
Unidentified
Proportion (%)
F
IgG Control (MaMET1)
MiMET1
MiMET1.1
MiMET1.2
Vimentin B220 S100A8 Ki67 PD-L1 DAPI
IgG Control
MiMet1.1
MiMet1.2
G
Vimentin
Ki67
PD-L1
MFI
0.0005
0.0010
<0.0001
<0.0001
0.0002
0.0008
H
Metastatic TIME
PD-L1
PD-1
Vim+CD44+Ki67+
Rechallenge
100% Metastatic Recurrence

Supplementary Figure 4. Combination treatment leads to ITH driven PD-1/PD-L1 expression in metastatic lung metastases. (A) Whole lung tissue H&E scans and IF staining of BrdU$^+$ tumor cells post 12-day T12 tumor cell TV injection, from an independent lung bearing metastases. (B) IHC staining of macrophage (F4/80) from whole lung tissues scans following IgG control or SNDX-ms6352, *(n = 3* independent mice*)*. (C) 20x magnification IHC staining of macrophage (F4/80) following IgG control or SNDX-ms6352. (D) Cumulative and individual marker (Vimentin, Pan-CK, CD8a, CD44, PD-1, FoxP3, F4/80, S100A8/9, PD-L1 and Ki67). IMC analysis was performed from 2 independently IgG treated lungs bearing macro-metastases depicted as control 1 and 2, n=3 ROI per lung. (E) Bar plots from IMC analysis of IgG control lung macro-metastases depicted as MaMets and combination treated lung micro-metastases depicted as MiMets looking for neutrophil, PanCK$^+$, PD-1$^+$, PD-L1$^+$ and tumor cell populations. (F) ITH is observed in combination treated MiMet1 and MiMet2 compared to MaMet1 IgG controls following IMC analysis (Vimentin, B220, S100A8, Ki67 and PD-L1). (G) IMC quantification of EMT (Vimentin), proliferation (Ki67) and exhaustion markers (PD-L1) in MiMet1.1, MiMet1.2 and IgG Control. Statistical analysis performed using two-way ANOVA with multiple comparisons test, (*n* = 3 mice). (H) Metastatic TIME was assessed via IMC. Data shown as mean ±SEM.

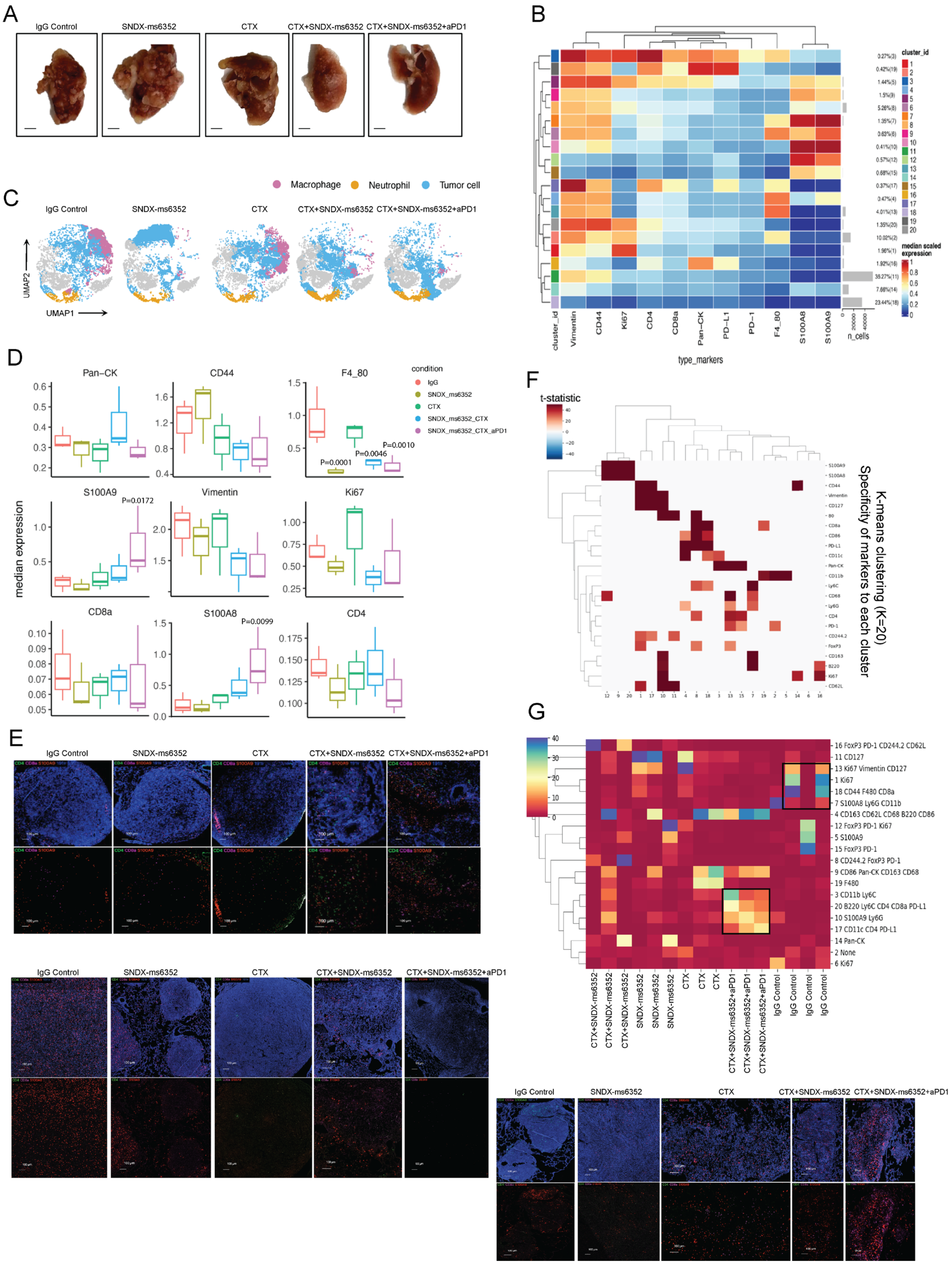
A
IgG Control
SNDX-ms6352
CTX
CTX+SNDX-ms6352
CTX+SNDX-ms6352+aPD1
B
cluster_id
Vimentin
CD44
Ki67
CD4
CD8a
Pan−CK
PD−L1
PD−1
F4_80
S100A8
S100A9
type_markers
n_cells
median scaled expression
C
Macrophage
Neutrophil
Tumor cell
UMAP1
UMAP2
D
median expression
Pan−CK
CD44
F4_80
S100A9
Vimentin
Ki67
CD8a
S100A8
CD4
condition
IgG
SNDX_ms6352
CTX
SNDX_ms6352_CTX
SNDX_ms6352_CTX_aPD1
P=0.0001
P=0.0046
P=0.0010
P=0.0172
P=0.0099
E
F
t-statistic
K-means clustering (K=20)
Specificity of markers to each cluster
G
16 FoxP3 PD-1 CD244.2 CD62L
11 CD127
13 Ki67 Vimentin CD127
1 Ki67
18 CD44 F480 CD8a
7 S100A8 Ly6G CD11b
4 CD163 CD62L CD68 B220 CD86
12 FoxP3 PD-1 Ki67
5 S100A9
15 FoxP3 PD-1
8 CD244.2 FoxP3 PD-1
9 CD86 Pan-CK CD163 CD68
19 F480
3 CD11b Ly6C
20 B220 Ly6C CD4 CD8a PD-L1
10 S100A9 Ly6G
17 CD11c CD4 PD-L1
14 Pan-CK
2 None
6 Ki67

Supplementary Figure 5. Addition of anti-PD1 reshapes the landscape of the metastatic TIME leading to the proximity of B-, T-cells and neutrophils and treatment response in established lung metastases. (A) Lung wholemounts from T12 IgG control, SNDX-ms6352, CTX and double or triple combination treated lungs, (*n = 5* mice per treatment). (B) Heatmap depicting 20 different clusters from individual protein markers (cluster_id) with the representative number of cells (n_cells) detected. (C) TSNE visualization of macrophage, neutrophil and tumor cells following IgG control, SNDX-ms6352, CTX and double or triple combination treated T12 treated lung metastases. (D) Median expression levels of PanCK, CD44, F4/80, S100A8/9, Ki67, CD4, CD8a and Vimentin following IgG control, SNDX-ms6352, CTX and double or triple combination treated T12 treated lung metastases. *P*-values were generated using diffcyt function (type of analysis, DS; method for DS testing, diffcyt-DS-limma). *P*-values were then adjusted using the Benjamini-Hochberg method, (*n* = 3 ROI). (E) IMC analysis of T cells (CD4 and CD8a) and neutrophils (S100A9) following IgG control, SNDX-ms6352, CTX and double or triple combination treated T12 treated lung metastases, (*n* = 3 ROI). (F) Unsupervised clustering of single cells segmented from IMC data from IgG control T12 lung metastases resulted in 20 clusters. (G) Expression levels of single cells segmented from IMC data from IgG control, SNDX-ms6352, CTX, CTX+SNDX-ms6352 and CTX+SNDX-ms6352+aPD-1 in T12 lung metastases from 20 clusters.

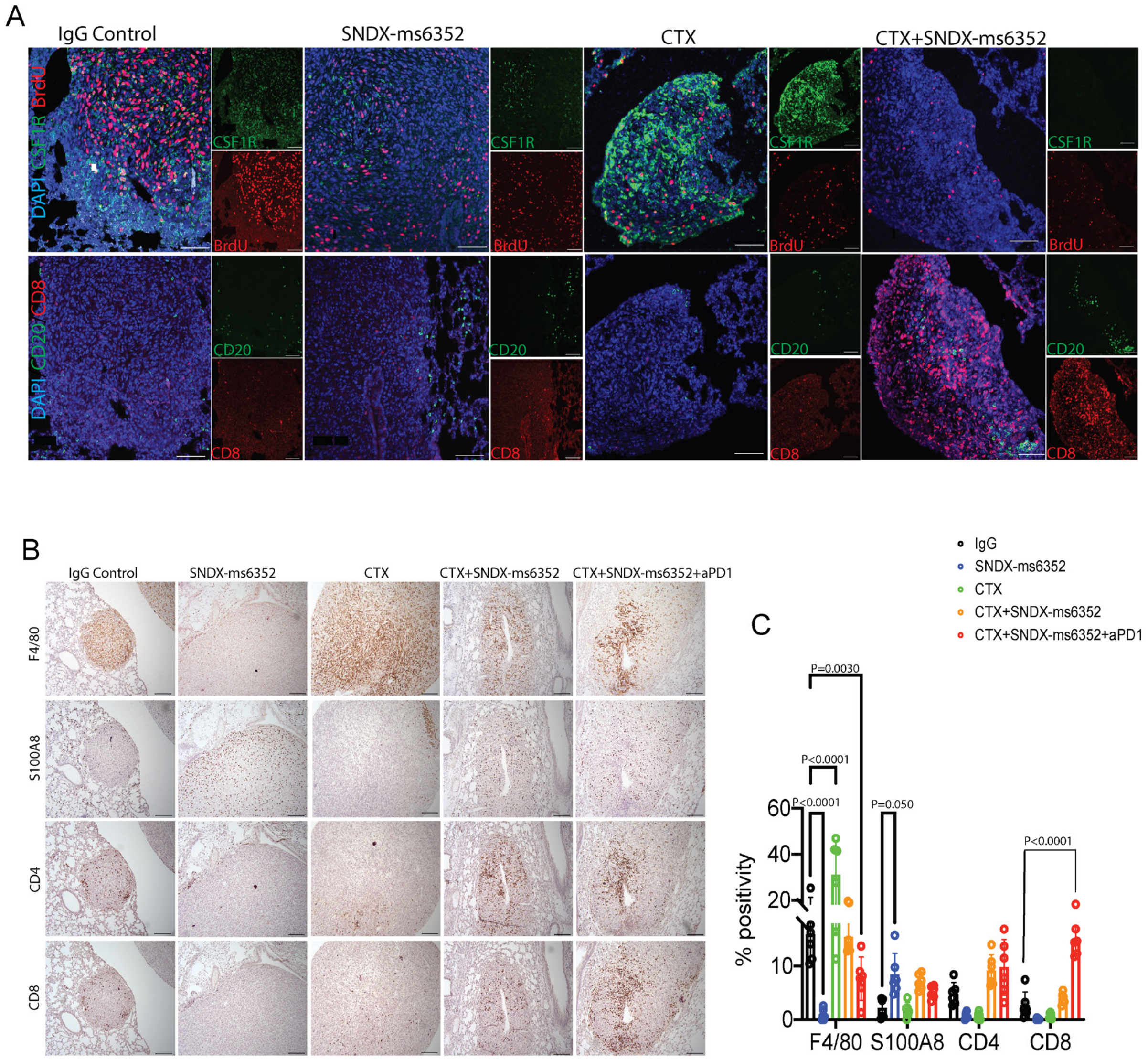
A
IgG Control
SNDX-ms6352
CTX
CTX+SNDX-ms6352
DAPI CSF1R BrdU
CSF1R
BrdU
DAPI CD20 CD8
CD20
CD8
B
IgG Control
SNDX-ms6352
CTX
CTX+SNDX-ms6352
CTX+SNDX-ms6352+aPD1
F4/80
S100A8
CD4
CD8
C
IgG
SNDX-ms6352
CTX
CTX+SNDX-ms6352
CTX+SNDX-ms6352+aPD1
P=0.0030
P<0.0001
P<0.0001
P=0.050
P<0.0001
% positivity
60
40
20
10
0
F4/80
S100A8
CD4
CD8

Supplementary Figure 6. Triple combination leads to long-term durable response of established lung metastases. (A) Double IF staining of CSF-1R, BrdU, CD20, CD8 and DAPI in T12 mice with established lung metastases following IgG control, SNDX-ms6352, CTX, CTX+SNDX-ms6352 or CTX+SNDX-ms6352+aPD-1, (*n = 5* mice per treatment). (B) IHC staining of macrophages (F4/80), neutrophils (S100A8) and T cells (CD4 and CD8) in T12 mice with established lung metastases following IgG control, SNDX-ms6352, CTX, CTX+SNDX-ms6352 or CTX+SNDX-ms6352+aPD-1, (*n = 5* mice per treatment). (C) IHC quantification of macrophages (F4/80), neutrophils (S100A8) and T cells (CD4 and CD8) from T12 mice with established lung metastases following IgG control, SNDX-ms6352, CTX, CTX+SNDX-ms6352 or CTX+SNDX-ms6352+aPD-1. Statistical analysis was performed using two-way ANOVA multiple comparisons test, (*n* = 5 mice per treatment). Data shown as mean ±SEM.

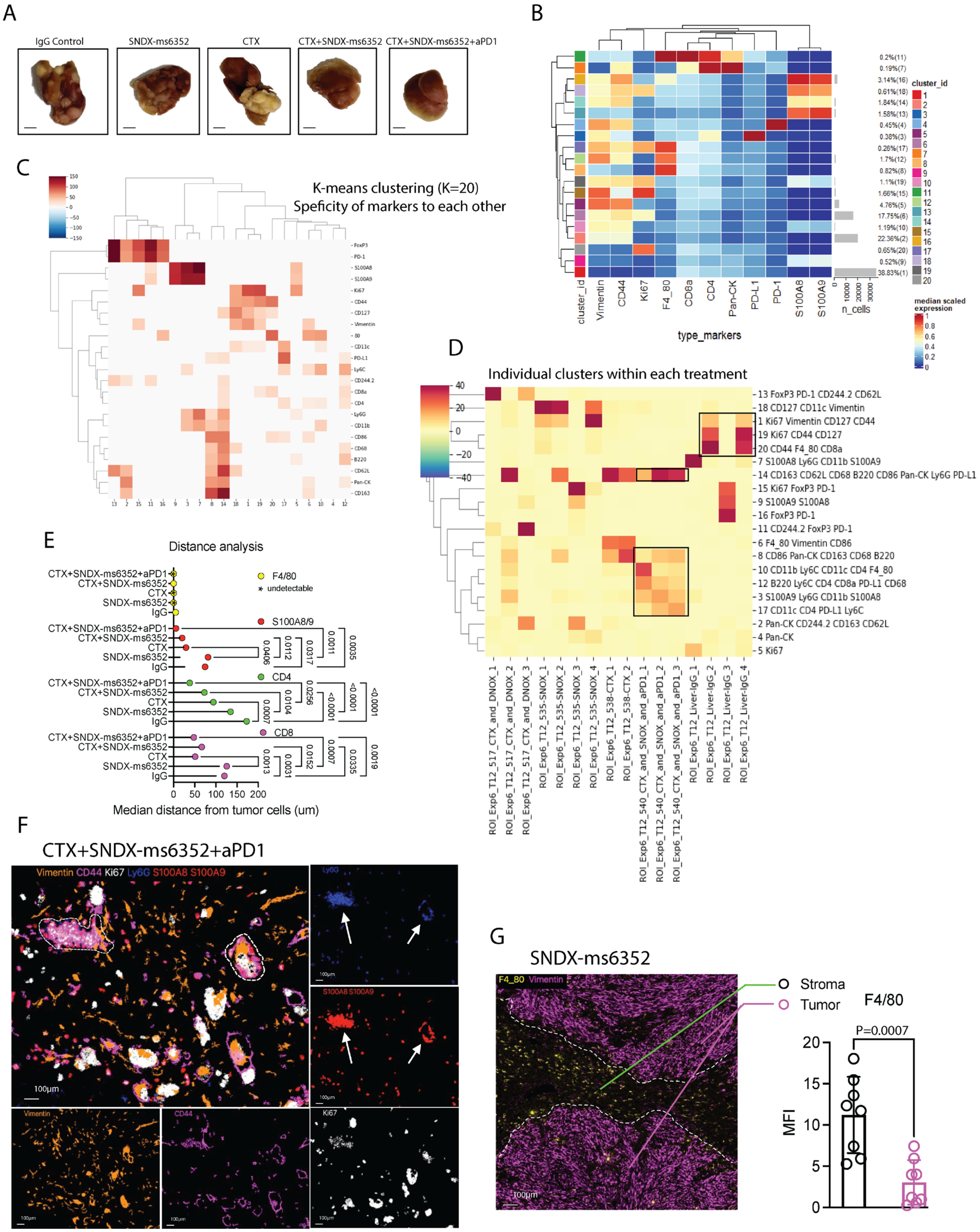
A
IgG Control
SNDX-ms6352
CTX
CTX+SNDX-ms6352
CTX+SNDX-ms6352+aPD1
B
cluster_id
type_markers
Vimentin
CD44
Ki67
F4_80
CD8a
CD4
Pan-CK
PD-L1
PD-1
S100A8
S100A9
n_cells
median scaled expression
C
K-means clustering (K=20)
Speficity of markers to each other
D
Individual clusters within each treatment
13 FoxP3 PD-1 CD244.2 CD62L
18 CD127 CD11c Vimentin
1 Ki67 Vimentin CD127 CD44
19 Ki67 CD44 CD127
20 CD44 F4_80 CD8a
7 S100A8 Ly6G CD11b S100A9
14 CD163 CD62L CD68 B220 CD86 Pan-CK Ly6G PD-L1
15 Ki67 FoxP3 PD-1
9 S100A9 S100A8
16 FoxP3 PD-1
11 CD244.2 FoxP3 PD-1
6 F4_80 Vimentin CD86
8 CD86 Pan-CK CD163 CD68 B220
10 CD11b Ly6C CD11c CD4 F4_80
12 B220 Ly6C CD4 CD8a PD-L1 CD68
3 S100A9 Ly6G CD11b S100A8
17 CD11c CD4 PD-L1 Ly6C
2 Pan-CK CD244.2 CD163 CD62L
4 Pan-CK
5 Ki67
E
Distance analysis
F4/80
undetectable
S100A8/9
CD4
CD8
CTX+SNDX-ms6352+aPD1
CTX+SNDX-ms6352
CTX
SNDX-ms6352
IgG
Median distance from tumor cells (um)
F
CTX+SNDX-ms6352+aPD1
Vimentin CD44 Ki67 Ly6G S100A8 S100A9
100µm
G
SNDX-ms6352
F4_80 Vimentin
Stroma
Tumor
F4/80
P=0.0007
MFI

Supplementary Figure 7. Established T12 liver metastases present with an immunologically cold TIME. (A) Liver wholemounts from T12 IgG control, SNDX-ms6352, CTX and double or triple combination treated lungs, (*n* = *3-5* mice per treatment). (B) Heatmap depicting 20 different clusters from individual protein markers with the representative number of cells. (C) Unsupervised clustering of single cells segmented from IMC data from IgG control T12 liver metastases resulted in 20 clusters. (D) Expression levels of single cells segmented from IMC data from IgG control, SNDX-ms6352, CTX, CTX+SNDX-ms6352 and CTX+SNDX-ms6352+aPD-1 in T12 lung metastases from 20 clusters. (E) Median distance analysis of immune cells from tumor cells post IgG, SNDX-ms6352, CTX, CTX+SNDX-ms6352 and CTX+SNDX-ms6352+aPD1 treated T12 syngeneic model at day 7, distance analysis is representative from IMC analysis of individual channels CD8, CD4, S100A8/9 and F4/80. Significance was determined using Two-way ANOVA and Tukey's multiple comparisons tests (*n* = 3 ROI). (F) Cumulative and individual marker (Vimentin, CD44, Ki67, Ly6G and S100A8/9) IMC analysis from multiple triple combination treated liver metastases. (*n* = 3 ROI). (G) IMC analysis and quantification of macrophages (F4/80) within the tumor bed and stroma of liver metastasis post SNDX-ms6352. Statistical analysis performed using paired t-test (*n* = 3 ROI). Data shown as mean ±SEM.

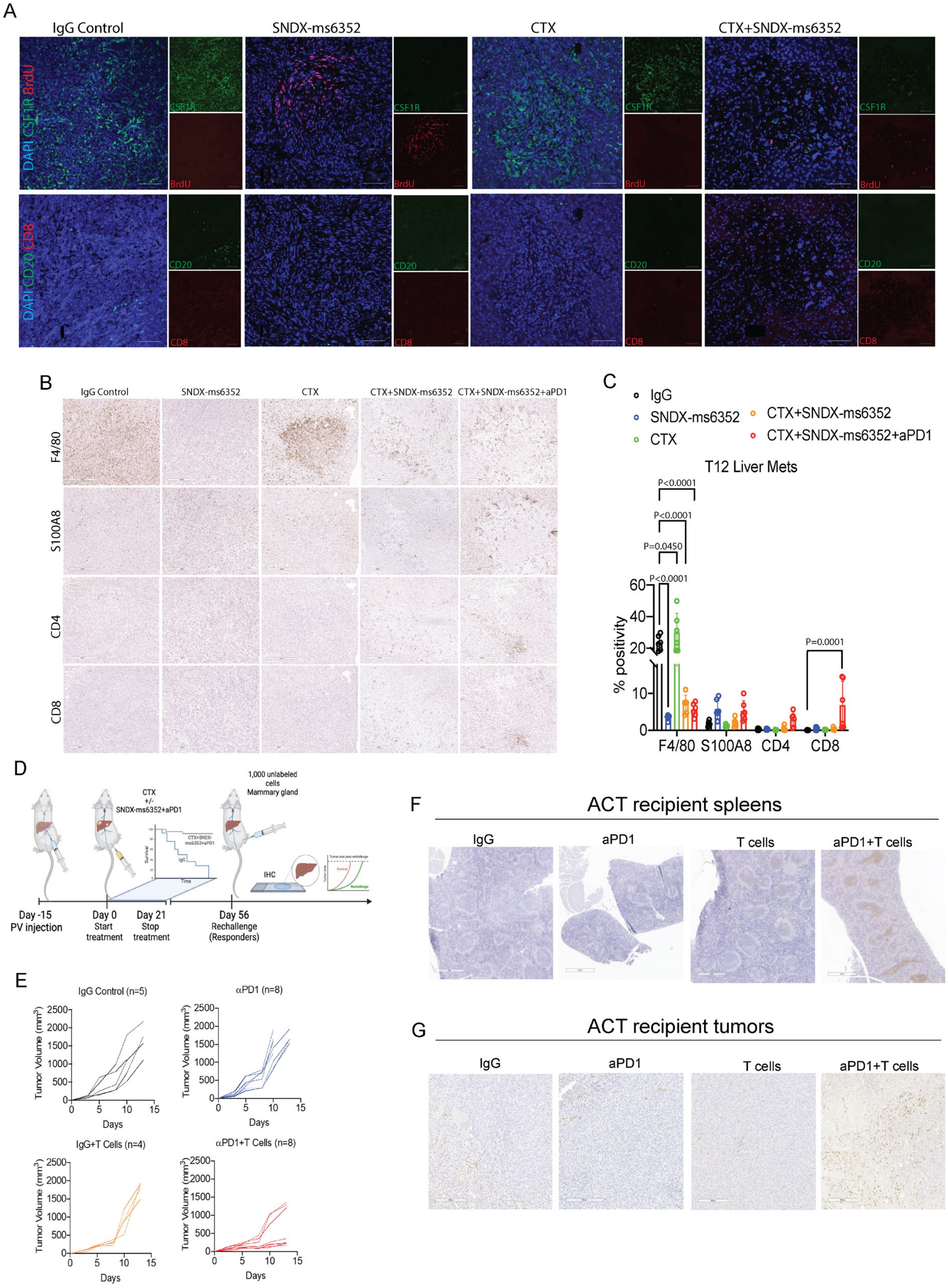
A
IgG Control
SNDX-ms6352
CTX
CTX+SNDX-ms6352
DAPI CSF1R BrdU
CSF1R
BrdU
DAPI CD20 CD8
CD20
CD8
B
IgG Control
SNDX-ms6352
CTX
CTX+SNDX-ms6352
CTX+SNDX-ms6352+aPD1
F4/80
S100A8
CD4
CD8
C
IgG
SNDX-ms6352
CTX
CTX+SNDX-ms6352
CTX+SNDX-ms6352+aPD1
T12 Liver Mets
P<0.0001
P<0.0001
P=0.0450
P<0.0001
P=0.0001
% positivity
F4/80
S100A8
CD4
CD8
D
CTX
+/-
SNDX-ms6352+aPD1
1,000 unlabeled cells
Mammary gland
IHC
Day -15
PV injection
Day 0
Start treatment
Day 21
Stop treatment
Day 56
Rechallenge (Responders)
E
IgG Control (n=5)
αPD1 (n=8)
IgG+T Cells (n=4)
αPD1+T Cells (n=8)
Tumor Volume (mm³)
Days
F
ACT recipient spleens
IgG
aPD1
T cells
aPD1+T cells
G
ACT recipient tumors
IgG
aPD1
T cells
aPD1+T cells

Supplementary Figure 8. An anti-PD-1 maintenance dose is necessary to generates long-term anti-tumor immunity and eradicate established liver metastases. (A) Double IF staining of CSF-1R, BrdU, CD20, CD8 and DAPI in T12 mice with established liver metastases following IgG control, SNDX-ms6352, CTX, CTX+SNDX-ms6352 (*n = 3* mice per treatment). (B) IHC staining of macrophages (F4/80), neutrophils (S100A8) and T cells (CD4 and CD8) in T12 mice with established liver metastases following IgG control, SNDX-ms6352, CTX, CTX+SNDX-ms6352 (*n = 3* mice per treatment. (C) IHC quantification of macrophages (F4/80), neutrophils (S100A8) and T cells (CD4 and CD8) from T12 mice with established lung metastases following IgG control, SNDX-ms6352, CTX, CTX+SNDX-ms6352. Statistical analysis was performed using two-way ANOVA multiple comparisons test, (*n = 3* mice per treatment). (D) Schematic representation of tumor cell rechallenge experiment. Post PV injection and treatment cessation at day 28, CR mice were rechallenged with 1,000 freshly dissociated tumor cells in the mammary gland. (E) Individual spider plots depicting tumor volume following IgG control, anti-PD-1, IgG control + T cells or anti-PD-1 + T cells from T12 tumor bearing mice following ACT. (F) IHC staining of CD8 T cells in spleen tissues following ACT (*n = 3* mice per treatment). (G) IHC staining of CD8 T cells in T12 primary tumors following ACT (*n = 3* mice per treatment). Data shown as mean ±SEM.

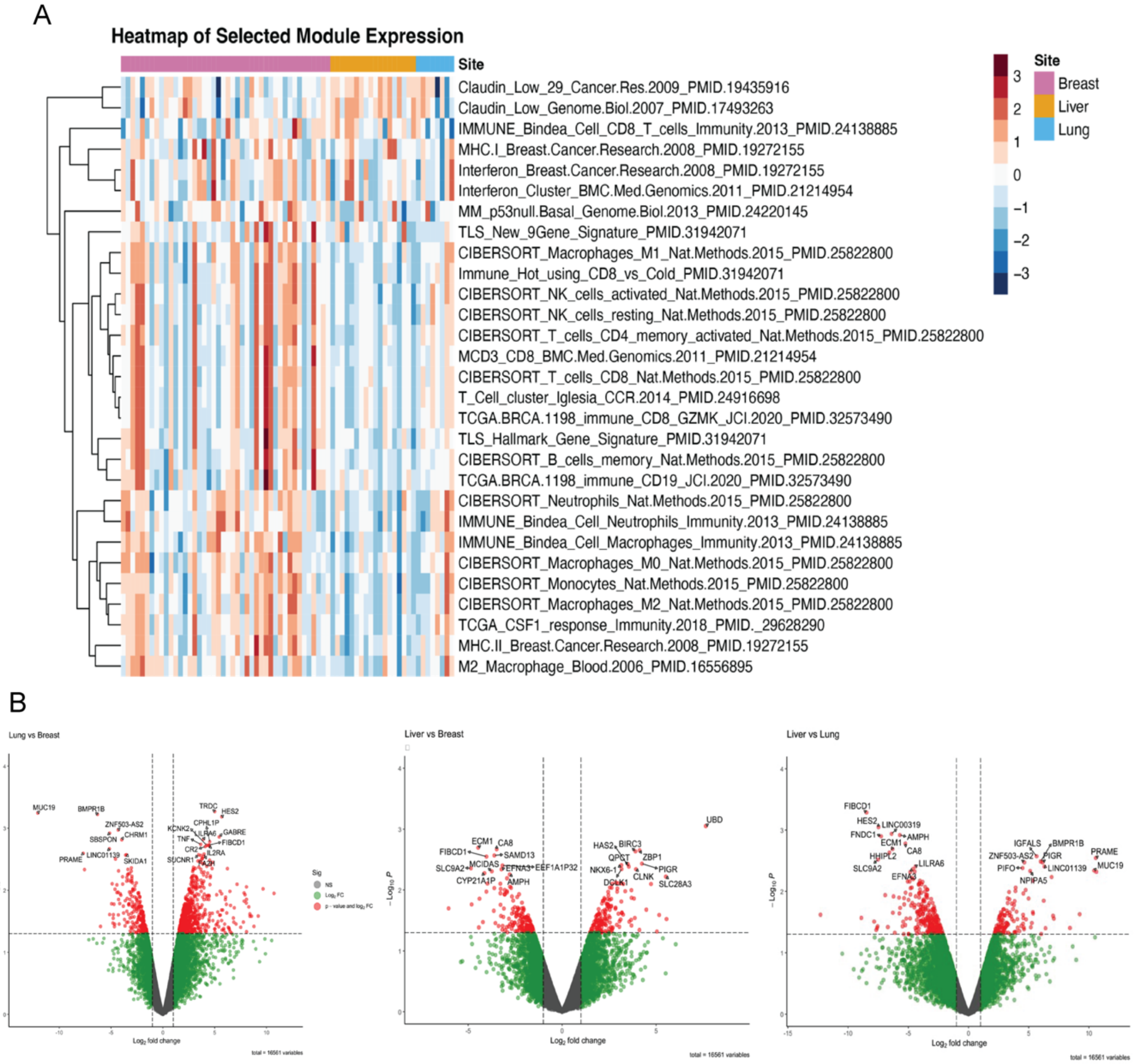

Supplementary Figure 9. Patients display elevated immunosuppressive macrophage signatures in lung and liver metastatic sites. (A) Representative heatmap of various immunological and claudin-low/p53 signatures between primary and metastatic tumor in multiple breast cancer subtypes (*n* = 44 primary versus *n* = 8 lung metastatic tumors and *n* = 18 liver metastatic tumors). (B) Volcano plot depicting the most up- and downregulated genes in metastatic samples comparing lung vs primary, liver vs breast and liver vs lung. For all multiple comparisons, p-values were adjusted using the Benjamini-Hochberg method. Gene set enrichment analyses were performed using the fgsea package. Gene sets with adjusted p-values < 0.05 were considered significant. Differential gene expression /expression signature analyses were performed using the limma package. Patient IDs were treated as a corresponding variable.

Supplementary Table 1

	H & E Staining Mice Tissue										
	Mice #	Slide #	<1 mm	1-3 mm	3-5 mm	>5 mm	*1	*2	*3	*4	Total score
Control	845	1-1	15	15	1	0	15	30	3	0	48
Control	845	2-1	18	18	2	0	18	36	6	0	60
Control	845	3-1	20	16	2	0	20	32	6	0	58
Control	845	4-1	14	17	0	0	14	34	0	0	48
Control	845	5-1	16	17	0	0	16	34	0	0	50
Control	850	1-1	16	16	2	2	16	32	6	8	62
Control	850	2-1	10	15	1	3	10	30	3	12	55
Control	850	3-1	15	14	0	3	15	28	0	12	55
Control	850	4-1	11	17	1	3	11	34	3	12	60
Control	850	5-1	16	23	2	2	16	46	6	8	76
Control	554	1-1	25	17	5	0	25	34	15	0	74
Control	554	2-1	25	17	4	0	25	34	12	0	71
Control	554	3-1	19	15	3	0	19	30	9	0	58
Control	554	4-1	24	20	1	0	24	40	3	0	67
Control	554	5-1	19	18	1	0	19	36	3	0	58
CTX	766	1-1	14	10	0	0	14	20	0	0	34
CTX	766	2-1	17	9	0	0	17	18	0	0	35
CTX	766	3-1	13	7	0	0	13	14	0	0	27
CTX	766	4-1	32	6	0	0	32	12	0	0	44
CTX	766	5-1	28	4	0	0	28	8	0	0	36
CTX	851	1-1	23	2	0	0	23	4	0	0	27
CTX	851	2-1	11	3	0	0	11	6	0	0	17
CTX	851	3-1	10	2	0	0	10	4	0	0	14
CTX	851	4-1	22	0	0	0	22	0	0	0	22
CTX	851	5-1	27	0	0	0	27	0	0	0	27
CTX	843	1-1	17	2	0	0	17	4	0	0	21
CTX	843	2-1	8	2	0	0	8	4	0	0	12
CTX	843	3-1	16	2	0	0	16	4	0	0	20
CTX	843	4-1	21	2	0	0	21	4	0	0	25
CTX	843	5-1	20	0	0	0	20	0	0	0	20
Double	759	1-1	6	2	0	0	6	4	0	0	10
Double	759	2-1	4	2	0	0	4	4	0	0	8
Double	759	3-1	4	0	0	0	4	0	0	0	4
Double	759	4-1	9	0	0	0	9	0	0	0	9
Double	759	5-1	3	0	0	0	3	0	0	0	3
Double	844	1-1	13	2	0	0	13	4	0	0	17
Double	844	2-1	18	0	0	0	18	0	0	0	18
Double	844	3-1	10	1	0	0	10	2	0	0	12
Double	844	4-1	9	2	0	0	9	4	0	0	13
Double	844	5-1	13	0	0	0	13	0	0	0	13
Double	769	1-1	21	4	0	0	21	8	0	0	29
Double	769	2-1	15	4	0	0	15	8	0	0	23
Double	769	3-1	19	1	0	0	19	2	0	0	21
Double	769	4-1	14	2	0	0	14	4	0	0	18
Double	769	5-1	11	0	0	0	11	0	0	0	11
SNDX	853	1-1	27	13	4	2	27	26	12	8	73
SNDX	853	2-1	27	16	2	2	27	32	6	8	73
SNDX	853	3-1	32	16	4	1	32	32	12	4	80
SNDX	853	4-1	36	16	2	1	36	32	6	4	78
SNDX	853	5-1	31	22	1	1	31	44	3	4	82
SNDX	770	1-1	26	11	2	0	26	22	6	0	54
SNDX	770	2-1	23	13	2	0	23	26	6	0	55
SNDX	770	3-1	30	17	2	0	30	34	6	0	70
SNDX	770	4-1	38	19	1	0	38	38	3	0	79
SNDX	770	5-1	43	16	1	0	43	32	3	0	78
SNDX	761	1-1	23	6	0	0	23	12	0	0	35
SNDX	761	2-1	16	9	0	0	16	18	0	0	34
SNDX	761	3-1	14	6	0	0	14	12	0	0	26
SNDX	761	4-1	19	3	0	0	19	6	0	0	25
SNDX	761	5-1	25	2	0	0	25	4	0	0	29
triple	669	1-1	2	0	0	0	2	0	0	0	2
triple	669	2-1	6	0	0	0	6	0	0	0	6
triple	669	3-1	9	0	0	0	9	0	0	0	9
triple	669	4-1	5	0	0	0	5	0	0	0	5
triple	669	5-1	4	1	0	0	4	2	0	0	6
triple	678	1-1	21	7	0	0	21	14	0	0	35
triple	678	2-1	18	4	0	0	18	8	0	0	26
triple	678	3-1	19	2	0	0	19	4	0	0	23
triple	678	4-1	16	4	0	0	16	8	0	0	24
triple	678	5-1	17	2	0	0	17	4	0	0	21
triple	762	1-1	9	1	0	0	9	2	0	0	11
triple	762	2-1	14	0	0	0	14	0	0	0	14
triple	762	3-1	11	0	0	0	11	0	0	0	11
triple	762	4-1	11	0	0	0	11	0	0	0	11
triple	762	5-1	16	0	0	0	16	0	0	0	16

Supplementary Table 2

	H & E Staining Mice Tissue										
	Mice #	Slide #	<1 mm	1-3 mm	3-5 mm	>5 mm	*1	*2	*3	*4	Total score
Double	517	1, 1	10	2	2		10	4	6	0	20
Double	517	2, 1	31	5	1	1	31	10	3	4	48
Double	517	3, 1	20	4		1	20	8	0	4	32
Double	517	4, 1	24	3		1	24	6	0	4	34
Double	517	5, 1	20	2	1		20	4	3	0	27
Double	516	1,1	19	2	1		19	4	3	0	26
Double	516	2,1	10	2	1		10	4	3	0	17
Double	516	3,1	8	4			8	8	0	0	16
Double	516	4,1	18	2			18	4	0	0	22
Double	516	5,1	11	2			11	4	0	0	15
Double	520	1,1	3				3	0	0	0	3
Double	520	2,1					0	0	0	0	0
Double	520	3,1	1				1	0	0	0	1
Double	520	4,1					0	0	0	0	0
Double	520	5,1					0	0	0	0	0
Triple	523	1,1	30	4	1		30	8	3	0	41
Triple	523	2,1	20	6	1		20	12	3	0	35
Triple	523	3,1	27	5	1		27	10	3	0	40
Triple	523	4,1	19	6			19	12	0	0	31
Triple	523	5,1	19	6			19	12	0	0	31
Control	541	1,1	9	5	2	4	9	10	6	16	41
Control	541	2,1	10	5	2	4	10	10	6	16	42
Control	541	3,1	11	5	2	4	11	10	6	16	43
Control	541	4,1	16	3	2	5	16	6	6	20	48
Control	541	5,1	14	8		4	14	16	0	16	46
Control	534	1,1	13	8	4	2	13	16	12	8	49
Control	534	2,1	17	8	4	2	17	16	12	8	53
Control	534	3,1	12	5	8	2	12	10	24	8	54
Control	534	4,1	18	7	4	2	18	14	12	8	52
Control	534	5,1	20	12	4	2	20	24	12	8	64
CTX	533	1,1	52	3		1	52	6	0	4	62
CTX	533	2,1	46	3		1	46	6	0	4	56
CTX	533	3,1	53	3		1	53	6	0	4	63
CTX	533	4,1	53	2		1	53	4	0	4	61
CTX	533	5,1	52	3		1	52	6	0	4	62
Triple	510	1,1	51	9		2	51	18	0	8	77
Triple	510	2,1	60	9		2	60	18	0	8	86
Triple	510	3,1	58	9	2	1	58	18	6	4	86
Triple	510	4,1	60	10	1	1	60	20	3	4	87
Triple	510	5,1	59	9	1	1	59	18	3	4	84
Control	514	1,1	22	8	1		22	16	3	0	41
Control	514	2,1	18	8	1		18	16	3	0	37
Control	514	3,1	21	7	1		21	14	3	0	38
Control	514	4,1	31	9	1		31	18	3	0	52
Control	514	5,1	49	8	1		49	16	3	0	68
Triple	518	1,1					0	0	0	0	0
Triple	518	2,1					0	0	0	0	0
Triple	518	3,1					0	0	0	0	0
Triple	518	4,1					0	0	0	0	0
Triple	518	5,1	1				1	0	0	0	1
CTX	513	1,1	6			1	6	0	0	4	10
CTX	513	2,1	5			1	5	0	0	4	9
CTX	513	3,1	3			1	3	0	0	4	7
CTX	513	4,1	6	1		1	6	2	0	4	12
CTX	513	5,1	5			1	5	0	0	4	9
SNDX	535	1,1	58	22		1	58	44	0	4	106
SNDX	535	2,1	53	26		1	53	52	0	4	109
SNDX	535	3,1	57	35			57	70	0	0	127
SNDX	535	4,1	56	35			56	70	0	0	126
SNDX	535	5,1	44	35		1	44	70	0	4	118
Triple	540	1,1	12	2			12	4	0	0	16
Triple	540	2,1	7	2			7	4	0	0	11
Triple	540	3,1	2	2			2	4	0	0	6
Triple	540	4,1	3	1			3	2	0	0	5
Triple	540	5,1	11	1			11	2	0	0	13
SNDX	529	1,1	26	4			26	8	0	0	34
SNDX	529	2,1	29	5			29	10	0	0	39
SNDX	529	3,1	24	5			24	10	0	0	34
SNDX	529	4,1	30	6			30	12	0	0	42
SNDX	529	5,1	26	7			26	14	0	0	40
CTX	538	1,1	25	11			25	22	0	0	47
CTX	538	2,1	30	8			30	16	0	0	46
CTX	538	3,1	23	13			23	26	0	0	49
CTX	538	4,1	22	13			22	26	0	0	48
CTX	538	5,1	25	6			25	12	0	0	37

Supplementary Table 3

	logFC	AveExpr	t	comp	P.Value	test			
CIBERSORT_B_cells_memory_Nat.Methods.2015_PMID.258228001	2.0026	0.869938	2.021058	Liver_vs_Breast	0.043451	1	0.043112	1	0.129337
CIBERSORT_B_cells_memory_Nat.Methods.2015_PMID.258228001	-0.00325	0.869938	-0.00232	Liver_vs_Lung	0.99815	1	0.043451	2	0.065177
CIBERSORT_B_cells_memory_Nat.Methods.2015_PMID.258228001	2.00585	0.869938	2.024338	Lung_vs_Breast	0.043112	1	0.99815	3	0.99815
CIBERSORT_Macrophages_M0_Nat.Methods.2015_PMID.258228001	0.81155	0.586817	0.81903	Liver_vs_Breast	0.412899	2	0.081175	1	0.243524
CIBERSORT_Macrophages_M0_Nat.Methods.2015_PMID.258228001	-0.9176	0.586817	-0.65482	Liver_vs_Lung	0.512683	2	0.412899	2	0.619349
CIBERSORT_Macrophages_M0_Nat.Methods.2015_PMID.258228001	1.72915	0.586817	1.745088	Lung_vs_Breast	0.081175	2	0.512683	3	0.512683
CIBERSORT_Macrophages_M1_Nat.Methods.2015_PMID.258228001	1.57065	0.737473	1.585127	Liver_vs_Breast	0.113149	3	0.036741	1	0.110224
CIBERSORT_Macrophages_M1_Nat.Methods.2015_PMID.258228001	-0.50076	0.737473	-0.35735	Liver_vs_Lung	0.720879	3	0.113149	2	0.169723
CIBERSORT_Macrophages_M1_Nat.Methods.2015_PMID.258228001	2.071405	0.737473	2.090498	Lung_vs_Breast	0.036741	3	0.720879	3	0.720879
CIBERSORT_Macrophages_M2_Nat.Methods.2015_PMID.258228001	1.212	0.511519	1.223171	Liver_vs_Breast	0.221457	4	0.090029	1	0.270086
CIBERSORT_Macrophages_M2_Nat.Methods.2015_PMID.258228001	-0.46885	0.511519	-0.33458	Liver_vs_Lung	0.737987	4	0.221457	2	0.332186
CIBERSORT_Macrophages_M2_Nat.Methods.2015_PMID.258228001	1.68085	0.511519	1.696343	Lung_vs_Breast	0.090029	4	0.737987	3	0.737987
CIBERSORT_Monocytes_Nat.Methods.2015_PMID.258228001	0.95325	0.467634	0.962036	Liver_vs_Breast	0.336187	5	0.200215	1	0.600645
CIBERSORT_Monocytes_Nat.Methods.2015_PMID.258228001	-0.31655	0.467634	-0.2259	Liver_vs_Lung	0.821312	5	0.336187	2	0.50428
CIBERSORT_Monocytes_Nat.Methods.2015_PMID.258228001	1.2698	0.467634	1.281504	Lung_vs_Breast	0.200215	5	0.821312	3	0.821312
CIBERSORT_Neutrophils_Nat.Methods.2015_PMID.258228001	0.6947	0.421166	0.701103	Liver_vs_Breast	0.483348	6	0.242782	1	0.728346
CIBERSORT_Neutrophils_Nat.Methods.2015_PMID.258228001	-0.46315	0.421166	-0.33052	Liver_vs_Lung	0.741057	6	0.483348	2	0.725021
CIBERSORT_Neutrophils_Nat.Methods.2015_PMID.258228001	1.15785	0.421166	1.168522	Lung_vs_Breast	0.242782	6	0.741057	3	0.741057
CIBERSORT_NK_cells_activated_Nat.Methods.2015_PMID.258228001	1.66642	0.779373	1.68178	Liver_vs_Breast	0.09282	7	0.091065	1	0.273196
CIBERSORT_NK_cells_activated_Nat.Methods.2015_PMID.258228001	-0.00903	0.779373	-0.00644	Liver_vs_Lung	0.994859	7	0.09282	2	0.13923
CIBERSORT_NK_cells_activated_Nat.Methods.2015_PMID.258228001	1.67545	0.779373	1.690893	Lung_vs_Breast	0.091065	7	0.994859	3	0.994859
CIBERSORT_NK_cells_resting_Nat.Methods.2015_PMID.258228001	1.754801	0.814159	1.770975	Liver_vs_Breast	0.076768	8	0.076768	1	0.230305
CIBERSORT_NK_cells_resting_Nat.Methods.2015_PMID.258228001	0.08365	0.814159	0.059695	Liver_vs_Lung	0.952406	8	0.091897	2	0.137846
CIBERSORT_NK_cells_resting_Nat.Methods.2015_PMID.258228001	1.67115	0.814159	1.686553	Lung_vs_Breast	0.091897	8	0.952406	3	0.952406
CIBERSORT_T_cells_CD4_memory_activated_Nat.Methods.2015_PMID.258228001	1.2046	0.740628	1.215703	Liver_vs_Breast	0.22429	9	0.025109	1	0.075326
CIBERSORT_T_cells_CD4_memory_activated_Nat.Methods.2015_PMID.258228001	-1.0169	0.740628	-0.72568	Liver_vs_Lung	0.468146	9	0.22429	2	0.336434
CIBERSORT_T_cells_CD4_memory_activated_Nat.Methods.2015_PMID.258228001	2.2215	0.740628	2.241976	Lung_vs_Breast	0.025109	9	0.468146	3	0.468146
CIBERSORT_T_cells_CD8_Nat.Methods.2015_PMID.258228001	1.6129	0.797469	1.627766	Liver_vs_Breast	0.103785	10	0.056651	1	0.169952
CIBERSORT_T_cells_CD8_Nat.Methods.2015_PMID.258228001	-0.27715	0.797469	-0.19778	Liver_vs_Lung	0.843243	10	0.103785	2	0.155677
CIBERSORT_T_cells_CD8_Nat.Methods.2015_PMID.258228001	1.89005	0.797469	1.907471	Lung_vs_Breast	0.056651	10	0.843243	3	0.843243
Claudin_Low_29_Cancer.Res.2009_PMID.194359161	-1.02681	-0.61163	-1.03627	Liver_vs_Breast	0.300244	11	0.300244	1	0.900731
Claudin_Low_29_Cancer.Res.2009_PMID.194359161	-1.06361	-0.61163	-0.75901	Liver_vs_Lung	0.447963	11	0.447963	2	0.671945
Claudin_Low_29_Cancer.Res.2009_PMID.194359161	0.0368	-0.61163	0.037139	Lung_vs_Breast	0.970379	11	0.970379	3	0.970379
Claudin_Low_Genome.Biol.2007_PMID.174932631	-1.8211	-0.7046	-1.83789	Liver_vs_Breast	0.066277	12	0.066277	1	0.198832
Claudin_Low_Genome.Biol.2007_PMID.174932631	-1.81845	-0.7046	-1.29769	Liver_vs_Lung	0.194594	12	0.194594	2	0.29189
Claudin_Low_Genome.Biol.2007_PMID.174932631	-0.00265	-0.7046	-0.00267	Lung_vs_Breast	0.997866	12	0.997866	3	0.997866
IMMUNE_Bindea_Cell_CD8_T_cells_Immunity.2013_PMID.241388851	0.68195	-0.33994	0.688236	Liver_vs_Breast	0.491411	13	0.491411	1	1.474233
IMMUNE_Bindea_Cell_CD8_T_cells_Immunity.2013_PMID.241388851	0.8178	-0.33994	0.583602	Liver_vs_Lung	0.559576	13	0.559576	2	0.839364
IMMUNE_Bindea_Cell_CD8_T_cells_Immunity.2013_PMID.241388851	-0.13585	-0.33994	-0.1371	Lung_vs_Breast	0.890968	13	0.890968	3	0.890968
IMMUNE_Bindea_Cell_Macrophages_Immunity.2013_PMID.241388851	0.30355	0.27607	0.306348	Liver_vs_Breast	0.759382	14	0.649033	1	1.9471
IMMUNE_Bindea_Cell_Macrophages_Immunity.2013_PMID.241388851	-0.14749	0.27607	-0.10525	Liver_vs_Lung	0.91619	14	0.759382	2	1.139074
IMMUNE_Bindea_Cell_Macrophages_Immunity.2013_PMID.241388851	0.45104	0.27607	0.455197	Lung_vs_Breast	0.649033	14	0.91619	3	0.91619
IMMUNE_Bindea_Cell_Neutrophils_Immunity.2013_PMID.241388851	0.78737	0.41264	0.794627	Liver_vs_Breast	0.426956	15	0.426956	1	1.280868
IMMUNE_Bindea_Cell_Neutrophils_Immunity.2013_PMID.241388851	0.002515	0.41264	0.001795	Liver_vs_Lung	0.998568	15	0.428434	2	0.642651
IMMUNE_Bindea_Cell_Neutrophils_Immunity.2013_PMID.241388851	0.784855	0.41264	0.792089	Lung_vs_Breast	0.428434	15	0.998568	3	0.998568
Immune_Hot_using_CD8_vs_Cold_PMID.319420711	1.6517	0.695877	1.666924	Liver_vs_Breast	0.095739	16	0.043373	1	0.130119
Immune_Hot_using_CD8_vs_Cold_PMID.319420711	-0.35165	0.695877	-0.25095	Liver_vs_Lung	0.80189	16	0.095739	2	0.143608
Immune_Hot_using_CD8_vs_Cold_PMID.319420711	2.00335	0.695877	2.021815	Lung_vs_Breast	0.043373	16	0.80189	3	0.80189
Interferon_Breast.Cancer.Research.2008_PMID.192721551	0.8682	0.143028	0.876202	Liver_vs_Breast	0.38106	17	0.38106	1	1.143181
Interferon_Breast.Cancer.Research.2008_PMID.192721551	0.34455	0.143028	0.245879	Liver_vs_Lung	0.805809	17	0.597247	2	0.89587
Interferon_Breast.Cancer.Research.2008_PMID.192721551	0.52365	0.143028	0.528477	Lung_vs_Breast	0.597247	17	0.805809	3	0.805809
Interferon_Cluster_BMC.Med.Genomics.2011_PMID.212149541	1.063	0.172957	1.072798	Liver_vs_Breast	0.283535	18	0.283535	1	0.850604
Interferon_Cluster_BMC.Med.Genomics.2011_PMID.212149541	0.62865	0.172957	0.44862	Liver_vs_Lung	0.653771	18	0.653771	2	0.980656
Interferon_Cluster_BMC.Med.Genomics.2011_PMID.212149541	0.43435	0.172957	0.438354	Lung_vs_Breast	0.661193	18	0.661193	3	0.661193
M2_Macrophage_Blood.2006_PMID.165568951	1.08433	0.080915	1.094325	Liver_vs_Breast	0.273989	19	0.273989	1	0.821966
M2_Macrophage_Blood.2006_PMID.165568951	0.25128	0.080915	0.17932	Liver_vs_Lung	0.857711	19	0.400634	2	0.600951
M2_Macrophage_Blood.2006_PMID.165568951	0.83305	0.080915	0.840728	Lung_vs_Breast	0.400634	19	0.857711	3	0.857711
MCD3_CD8_BMC.Med.Genomics.2011_PMID.212149541	1.80695	0.778527	1.823605	Liver_vs_Breast	0.068411	20	0.036062	1	0.108187
MCD3_CD8_BMC.Med.Genomics.2011_PMID.212149541	-0.272	0.778527	-0.19411	Liver_vs_Lung	0.846119	20	0.068411	2	0.102616
MCD3_CD8_BMC.Med.Genomics.2011_PMID.212149541	2.07895	0.778527	2.098112	Lung_vs_Breast	0.036062	20	0.846119	3	0.846119
MHC.I_Breast.Cancer.Research.2008_PMID.192721551	0.69	0.289022	0.69636	Liver_vs_Breast	0.486312	21	0.037175	1	0.111524
MHC.I_Breast.Cancer.Research.2008_PMID.192721551	-1.37665	0.289022	-0.98241	Liver_vs_Lung	0.326056	21	0.326056	2	0.489084
MHC.I_Breast.Cancer.Research.2008_PMID.192721551	2.06665	0.289022	2.085699	Lung_vs_Breast	0.037175	21	0.486312	3	0.486312
MHC.II_Breast.Cancer.Research.2008_PMID.192721551	0.986	0.348245	0.995088	Liver_vs_Breast	0.319854	22	0.031042	1	0.093127
MHC.II_Breast.Cancer.Research.2008_PMID.192721551	-1.15285	0.348245	-0.8227	Liver_vs_Lung	0.410809	22	0.319854	2	0.479781
MHC.II_Breast.Cancer.Research.2008_PMID.192721551	2.13885	0.348245	2.158564	Lung_vs_Breast	0.031042	22	0.410809	3	0.410809
MM_p53null.Basal_Genome.Biol.2013_PMID.242201451	-0.15765	0.111895	-0.1591	Liver_vs_Breast	0.873609	23	0.540905	1	1.622714
MM_p53null.Basal_Genome.Biol.2013_PMID.242201451	-0.76365	0.111895	-0.54496	Liver_vs_Lung	0.585863	23	0.585863	2	0.878794
MM_p53null.Basal_Genome.Biol.2013_PMID.242201451	0.606	0.111895	0.611586	Lung_vs_Breast	0.540905	23	0.873609	3	0.873609
T_Cell_cluster_Iglesia_CCR.2014_PMID.249166981	1.54825	0.775095	1.562521	Liver_vs_Breast	0.118377	24	0.043009	1	0.129026
T_Cell_cluster_Iglesia_CCR.2014_PMID.249166981	-0.4586	0.775095	-0.32727	Liver_vs_Lung	0.743511	24	0.118377	2	0.177565
T_Cell_cluster_Iglesia_CCR.2014_PMID.249166981	2.00685	0.775095	2.025348	Lung_vs_Breast	0.043009	24	0.743511	3	0.743511
TCGA.BRCA.1198_immune_CD19_JCI.2020_PMID.325734901	1.7631	0.901708	1.779351	Liver_vs_Breast	0.075385	25	0.057498	1	0.172494
TCGA.BRCA.1198_immune_CD19_JCI.2020_PMID.325734901	-0.12051	0.901708	-0.086	Liver_vs_Lung	0.931482	25	0.075385	2	0.113077
TCGA.BRCA.1198_immune_CD19_JCI.2020_PMID.325734901	1.883605	0.901708	1.900967	Lung_vs_Breast	0.057498	25	0.931482	3	0.931482
TCGA.BRCA.1198_immune_CD8_GZMK_JCI.2020_PMID.325734901	1.5748	0.765913	1.589315	Liver_vs_Breast	0.1122	26	0.043603	1	0.13081
TCGA.BRCA.1198_immune_CD8_GZMK_JCI.2020_PMID.325734901	-0.42635	0.765913	-0.30425	Liver_vs_Lung	0.760977	26	0.1122	2	0.1683
TCGA.BRCA.1198_immune_CD8_GZMK_JCI.2020_PMID.325734901	2.00115	0.765913	2.019595	Lung_vs_Breast	0.043603	26	0.760977	3	0.760977
TCGA_CSF1_response_Immunity.2018_PMID._296282901	1.14575	0.565465	1.156311	Liver_vs_Breast	0.247738	27	0.150462	1	0.451385
TCGA_CSF1_response_Immunity.2018_PMID._296282901	-0.27975	0.565465	-0.19964	Liver_vs_Lung	0.841792	27	0.247738	2	0.371608
TCGA_CSF1_response_Immunity.2018_PMID._296282901	1.4255	0.565465	1.438639	Lung_vs_Breast	0.150462	27	0.841792	3	0.841792
TLS_Hallmark_Gene_Signature_PMID.319420711	1.27495	0.621337	1.286702	Liver_vs_Breast	0.198397	28	0.019413	1	0.05824
TLS_Hallmark_Gene_Signature_PMID.319420711	-1.0437	0.621337	-0.74481	Liver_vs_Lung	0.456504	28	0.198397	2	0.297596
TLS_Hallmark_Gene_Signature_PMID.319420711	2.31865	0.621337	2.340022	Lung_vs_Breast	0.019413	28	0.456504	3	0.456504
TLS_New_9Gene_Signature_PMID.319420711	1.48605	0.645191	1.499747	Liver_vs_Breast	0.133891	29	0.133891	1	0.401672
TLS_New_9Gene_Signature_PMID.319420711	0.6166	0.645191	0.440021	Liver_vs_Lung	0.659986	29	0.380375	2	0.570563
TLS_New_9Gene_Signature_PMID.319420711	0.86945	0.645191	0.877464	Lung_vs_Breast	0.380375	29	0.659986	3	0.659986

Supplementary Table 4

Diego/Rosen Mouse FFPE IMC panel

#	Marker	Clone	Metal	Vendor	Catlog#	AR condition	Final Dilution (1:XX)
7	CD11B	EPR1344	113Cd	Abcam	ab209970	Tris EDTA, pH9.0/Steamer, 30min	1:100
11	Ly6G	1A8	141Pr	BioLegend	127602	Tris EDTA, pH9.0/Steamer, 30min	1:50
21	Vimentin	D21H3	143Nd	Cell Signaling	4617SF	Tris EDTA, pH9.0/Steamer, 30min	1:1000
8	PAN-CK	AE-1/AE-3	144Nd	BioLegend	914204	Tris EDTA, pH9.0/Steamer, 30min	1:50
19	CD163	POLY	145Nd	ThermoFisher	16646-1-AP	Tris EDTA, pH9.0/Steamer, 30min	1:50
1	CD4	BLR167J	146Nd	Bethyl	A700-167	Tris EDTA, pH9.0/Steamer, 30min	1:50
2	CD8	4SM16	147Sm	Fisher Scientific	14-019-537	Tris EDTA, pH9.0/Steamer, 30min	1:50
15	CD127	EPR2955(2)	149Sm	Abcam	ab240225	Tris EDTA, pH9.0/Steamer, 30min	1:100
6	CD11C	D1V9Y	151Eu	Cell Signaling	39143SF	Tris EDTA, pH9.0/Steamer, 30min	1:50
17	CD44	IM7	153Eu	BioLegend	103002	Tris EDTA, pH9.0/Steamer, 30min	1:50
18	CD244.2	M2B4	154Sm	BioLegend	133501	Tris EDTA, pH9.0/Steamer, 30min	1:50
13	PD1	12A7D7	155Gd	ThermoFisher	MA515781	Tris EDTA, pH9.0/Steamer, 30min	1:50
10	FOXP3	FJK-16s	158Gd	ThermoFisher	14-5773-82	Tris EDTA, pH9.0/Steamer, 30min	1:50
3	F4/80	D2S9R	159Tb	Cell Signaling	25514SF	Tris EDTA, pH9.0/Steamer, 30min	1:50
16	CD62L	RB POLY	160Gd	ThermoFisher	PA5-595721	Tris EDTA, pH9.0/Steamer, 30min	1:50
5	CD86	E5W6H	161Dy	Cell Signaling	19589BF	Tris EDTA, pH9.0/Steamer, 30min	1:50
22	S100A8	E4F8V	163Dy	Cell Signaling	47310S	Tris EDTA, pH9.0/Steamer, 30min	1:1000
23	S100A9	D3U8M	164Dy	Cell Signaling	73425BF	Tris EDTA, pH9.0/Steamer, 30min	1:1000
14	PD-L1	D5V3B	165Ho	Cell Signaling	64988BF	Tris EDTA, pH9.0/Steamer, 30min	1:50
9	Ki-67	8D5	168Er	Novus	NBP2-22112	Tris EDTA, pH9.0/Steamer, 30min	1:100
20	CD68	POLY	171Yb	ThermoFisher	PA589134	Tris EDTA, pH9.0/Steamer, 30min	1:100
12	Ly6C	ER-MP20	173Yb	Novus	NB100-65413	Tris EDTA, pH9.0/Steamer, 30min	1:50
4	B220	RA3-6B2	176Yb	BioLegend	103202	Tris EDTA, pH9.0/Steamer, 30min	1:50
