# Supplementary material for: Anti-CSF-1R therapy with combined immuno-chemotherapy coordinate an adaptive immune response to eliminate macrophage enriched triple negative breast cancers
Source: Nat Commun. 2026 Jan 3;17:1193. doi: 10.1038/s41467-025-67964-2 (PMC12858953; doi:10.1038/s41467-025-67964-2)
Supplement: Supplementary file 2 — Description of Additional Supplementary Files [file 41467_2025_67964_MOESM2_ESM.pdf]

Description of Additional Supplementary Files

Supplementary dataset 1: m0_GTPase-TAM_DEG_sig

Supplementary dataset 2: m1_LA-TAM-1_DEG_sig Supplementary dataset 3: m2_Res-TAM_DEG_sig

Supplementary dataset 4: m3_Inflam-TAM_DEG_sig

Supplementary dataset 5: m4_LA-TAM-2_DEG_sig

Supplementary dataset 6: m5_Monocyte_DEG_sig

Supplementary dataset 7: m6_Reg-TAM_DEG_sig

Supplementary dataset 8: m7_Prolif-TAM_DEG_sig

Supplementary dataset 9: m8_Prolif-TAM_DEG_sig S

Supplementary dataset 10: m_inflam_GO

Supplementary dataset 11: Residual lipid TAM GO

Supplementary dataset 12: Raw Cytokine levels normalized to controls from plasma post treated T12 mice bearing primary tumors.

Supplementary dataset 13: Raw Cytokine levels from tumor lysates normalized to controls from post treated T12 mice bearing primary tumors.

Supplementary dataset 14: AURORA Cibersort sig_genes_TNBC_met_vs_primary.

Supplementary dataset 15: Single-cell level counts from IMC from merged treated lungs.

Supplementary dataset 16: Single-cell level counts from IMC from merged treated lungs**.**

Supplementary dataset 17: Single-cell level counts from IMC from merged treated livers.
