# Supplementary material for: Anti-CSF-1R therapy with combined immuno-chemotherapy coordinate an adaptive immune response to eliminate macrophage enriched triple negative breast cancers
Source: Nat Commun. 2026 Jan 3;17:1193. doi: 10.1038/s41467-025-67964-2 (PMC12858953; doi:10.1038/s41467-025-67964-2)
Supplement: Supplementary file 20 — Reporting Summary [file 41467_2025_67964_MOESM20_ESM.pdf]

nature portfolio

Corresponding author(s): Jeffrey M. Rosen

Last updated by author(s): Oct 23, 2025

Reporting Summary

Nature Portfolio wishes to improve the reproducibility of the work that we publish. This form provides structure for consistency and transparency in reporting. For further information on Nature Portfolio policies, see our Editorial Policies and the Editorial Policy Checklist.

Statistics

For all statistical analyses, confirm that the following items are present in the figure legend, table legend, main text, or Methods section.

n/a	Confirmed	
☐	☒	The exact sample size (*n*) for each experimental group/condition, given as a discrete number and unit of measurement
☐	☒	A statement on whether measurements were taken from distinct samples or whether the same sample was measured repeatedly
☐	☒	The statistical test(s) used AND whether they are one- or two-sided<br>*Only common tests should be described solely by name; describe more complex techniques in the Methods section.*
☐	☒	A description of all covariates tested
☐	☒	A description of any assumptions or corrections, such as tests of normality and adjustment for multiple comparisons
☐	☒	A full description of the statistical parameters including central tendency (e.g. means) or other basic estimates (e.g. regression coefficient) AND variation (e.g. standard deviation) or associated estimates of uncertainty (e.g. confidence intervals)
☐	☒	For null hypothesis testing, the test statistic (e.g. *F*, *t*, *r*) with confidence intervals, effect sizes, degrees of freedom and *P* value noted<br>*Give P values as exact values whenever suitable.*
☒	☐	For Bayesian analysis, information on the choice of priors and Markov chain Monte Carlo settings
☐	☒	For hierarchical and complex designs, identification of the appropriate level for tests and full reporting of outcomes
☒	☐	Estimates of effect sizes (e.g. Cohen's *d*, Pearson's *r*), indicating how they were calculated

Our web collection on statistics for biologists contains articles on many of the points above.

Software and code

Policy information about availability of computer code

Data collection	IMC imaging data was collected using the the Hyperion imaging system. For scRNA-seq Viable single cell suspensions were tagged with 3' CellPlex barcoding oligos (10X Genomics, PN-1000262), Single-cell libraries were prepared following the Chromium Single Cell Gene Expression 3' v3.1 kit encompassing the multiplex capture CMO barcoding kit (10x Genomics, PN-1000262). Next generation library sequencing was performed using the NovaSeq 6000 (Illumina) following quality control check.
Data analysis	For IMC, Cell-level image quality-control is first evaluated with the MCD viewer software provided by Standard Biotools. The full resolution IMC images (10X) in MCD. format is added to the Visiopharm® database and color-adjusted for best visualization for nuclear detection and segmentation. Cell segmentation is performed using DNA signal to find the nuclei followed by Visiopharm® designated algorithms including polynomial blobs to refine the separation of cells. Analyzed data with all cells and their phenotypes were exported to TSV. file for further analysis. TSV files were first processed in R using FlowSOM, flowCore and SingleCellExperiment packages in preparation for further analyses using the CATALYST package (version 1.14.0, https://github.com/HelenaLC/CATALYST). Image acquisition was performed using the available histoCAT web (http://github.com/BodenmillerGroup/histocat-web) software. For scRNA-seq, the raw sequencing data, alignment, read counts and sample demultiplexing was performed using the CellRanger multi (v7.2.0) pipeline. The barcode assignment confidence was set to 0.8 to capture a larger number of cells for downstream processing. scRNA-seq was analyzed using the Seurat (v4.4.0) package in R (R version 4.3.1; https://github.com/satijalab/seurat). The PET images were quantified using Inveon Research Workspace IRW (IRW, Siemens AG, Knoxville, TN). Graph-pad prism 9 was used for figure creation and basic statistical analysis.

For manuscripts utilizing custom algorithms or software that are central to the research but not yet described in published literature, software must be made available to editors and reviewers. We strongly encourage code deposition in a community repository (e.g. GitHub). See the Nature Portfolio guidelines for submitting code & software for further information.

Data

Policy information about availability of data

All manuscripts must include a data availability statement. This statement should provide the following information, where applicable:

- Accession codes, unique identifiers, or web links for publicly available datasets
- A description of any restrictions on data availability
- For clinical datasets or third party data, please ensure that the statement adheres to our policy

Raw and analyzed scRNA-seq data has been deposited in the NCBI GEO database accession number (GSE292908). The batch-corrected and normal tissue-adjusted gene expression and expression signature data were obtained from the AURORA USA clinical study (GSE209998). For primary figures the data were filtered to contain only triple-negative primary tumors, lung metastases, and liver metastases (Supplementary tables S16 and S17).Detailed information regarding the antibodies used for IMC is provided in Supplementary table S18, IMC single cell analysis provided in Supplementary tables S19-21.

Research involving human participants, their data, or biological material

Policy information about studies with human participants or human data. See also policy information about sex, gender (identity/presentation), and sexual orientation and race, ethnicity and racism.

Reporting on sex and gender	*Use the terms sex (biological attribute) and gender (shaped by social and cultural circumstances) carefully in order to avoid confusing both terms. Indicate if findings apply to only one sex or gender; describe whether sex and gender were considered in study design; whether sex and/or gender was determined based on self-reporting or assigned and methods used. Provide in the source data disaggregated sex and gender data, where this information has been collected, and if consent has been obtained for sharing of individual-level data; provide overall numbers in this Reporting Summary. Please state if this information has not been collected. Report sex- and gender-based analyses where performed, justify reasons for lack of sex- and gender-based analysis.*
Reporting on race, ethnicity, or other socially relevant groupings	*Please specify the socially constructed or socially relevant categorization variable(s) used in your manuscript and explain why they were used. Please note that such variables should not be used as proxies for other socially constructed/relevant variables (for example, race or ethnicity should not be used as a proxy for socioeconomic status). Provide clear definitions of the relevant terms used, how they were provided (by the participants/respondents, the researchers, or third parties), and the method(s) used to classify people into the different categories (e.g. self-report, census or administrative data, social media data, etc.) Please provide details about how you controlled for confounding variables in your analyses.*
Population characteristics	*Describe the covariate-relevant population characteristics of the human research participants (e.g. age, genotypic information, past and current diagnosis and treatment categories). If you filled out the behavioural & social sciences study design questions and have nothing to add here, write "See above."*
Recruitment	*Describe how participants were recruited. Outline any potential self-selection bias or other biases that may be present and how these are likely to impact results.*
Ethics oversight	*Identify the organization(s) that approved the study protocol.*

Note that full information on the approval of the study protocol must also be provided in the manuscript.

Field-specific reporting

Please select the one below that is the best fit for your research. If you are not sure, read the appropriate sections before making your selection.

☒ Life sciences ☐ Behavioural & social sciences ☐ Ecological, evolutionary & environmental sciences

For a reference copy of the document with all sections, see nature.com/documents/nr-reporting-summary-flat.pdf

Life sciences study design

All studies must disclose on these points even when the disclosure is negative.

Sample size	No calculations were done to determine sample size. Sample size was determined based on standards for experimental cancer cell biology and animal studies, attempting to have a minimum of N = 3 biological replicates with sufficient reproducibility.
Data exclusions	Several end points for mice were excluded from Figure 4B at the request of reviewers, these mice grew tumors on the tails following tail vein injections. Thus we omitted them from the analyses, this has been discussed in the manuscript.
Replication	All experimental findings were replicated at least 3 times with enough reproducibility.
Randomization	Allocation of samples was random.
Blinding	For animal treatment experiments, investigators were not blinded to group allocation during data collection due to limited lab personnel.

Reporting for specific materials, systems and methods

We require information from authors about some types of materials, experimental systems and methods used in many studies. Here, indicate whether each material, system or method listed is relevant to your study. If you are not sure if a list item applies to your research, read the appropriate section before selecting a response.

Materials & experimental systems

n/a	Involved in the study
☐	☒ Antibodies
☐	☒ Eukaryotic cell lines
☐	☐ Palaeontology and archaeology
☐	☒ Animals and other organisms
☒	☐ Clinical data
☒	☐ Dual use research of concern
☒	☐ Plants

Methods

n/a	Involved in the study
☒	☐ ChIP-seq
☐	☒ Flow cytometry
☒	☐ MRI-based neuroimaging

Antibodies

Antibodies used

For immunoblotting: CSF-1R (Cell Signaling Technology, 43390) and CD206/MRC1 (Cell Signaling Technology, 24595)
For IHC and IF: F4/80 (Cell Signaling Technology, 70076; 1:500), S100A8 (R&D Systems, MAB3059; 1:5000), CD8α (Cell Signaling Technology, 98941; 1:500) and CD4 (Abcam, ab183685 1:400), BrdU (1:1000; Abcam, ab6326), cleaved caspase 3 (Cell Signaling Technology, 9661, 1:1000), CD20 (Cell Signaling Technology, 70168; 1:500), anti-CSF-1R/M-CSF-R (Cell Signaling Technology, 43390, 1:500) and anti-CD8α (eBioscience, 14-0081-82; 1:500).
For in vivo and in vitro treatments: CSF-1R monoclonal antibody, SNDX-ms6352 was donated by Syndax Pharmaceuticals, Inc. InVivoMab mouse IgG1 isotype control (Bio X Cell, BE0083), RecombiMAb anti-mouse PD1 (BioXcell, CD279, CP151) and RecombiMAb anti-mouse IgG2a isotype (BioXcell, CP150).
Detailed information regarding the antibodies used for IMC is provided in Supplementary table S18.

Validation

Antibodies used for immunoblotting, IHC or IF have been cited. For example, CD8 PMID:40180890, CD4 PMID:37874652, BrdU PMID: 38123565, Cleaved Caspase 3 PMID:40188189, CD20 PMID: 39994401.
CSF-1R used in immunoblotting and IF assay have been validated by macrophage SNDX-ms6352 depleting antibody and appropriate IgG control treatments.
For IMC: For antibody validation a separate tissue microarray (TMA) was generated using normal spleen, normal lung, lung metastases and primary tumor tissues from paraffin embedded blocks, sectioned at 5 µm. Each specific region of the slide contained the four varying tissues and was separated using a hydrophobic PAP pen. The TMA was generated by the Human Tissue Acquisition and Pathology Core and inspected by a pathologist to assess tissues. Four different antibody cocktail dilutions were then tested, each cocktail contained 23 different antibodies. Multiple antigen retrieval buffers were simultaneously tested with each cocktail dilution, including citrate buffer (pH 6.0) and IHC Antigen Retrieval Solution (10 mM Tris, 1 mM EDTA, pH 9.0) (eBioscience, 00-4956-58). The different cocktail groups were obtained analyzed and validated in conjunction with The Cytometry and Cell Sorting Core (CCSC) at Baylor College of Medicine (BCM).

Eukaryotic cell lines

Policy information about cell lines and Sex and Gender in Research

Cell line source(s)

Primary cell lines were generated from genetically engineered mouse (GEM) mammary tumors. All of these GEMMs have been genomically and histologically characterized.

Authentication

None of the cell lines used were authenticated.

Mycoplasma contamination

All cells were tested to be free of mycoplasma contaminants using the Universal Mycoplasma Detection Kit (ATCC, 30-1012K).

Commonly misidentified lines
(See ICLAC register)

No commonly misidentified cell lines were used in the study.

Palaeontology and Archaeology

Specimen provenance

Provide provenance information for specimens and describe permits that were obtained for the work (including the name of the issuing authority, the date of issue, and any identifying information). Permits should encompass collection and, where applicable, export.

Specimen deposition

Indicate where the specimens have been deposited to permit free access by other researchers.

Dating methods

If new dates are provided, describe how they were obtained (e.g. collection, storage, sample pretreatment and measurement), where

Dating methods — they were obtained (i.e. lab name), the calibration program and the protocol for quality assurance OR state that no new dates are provided.

☐ Tick this box to confirm that the raw and calibrated dates are available in the paper or in Supplementary Information.

Ethics oversight — Identify the organization(s) that approved or provided guidance on the study protocol, OR state that no ethical approval or guidance was required and explain why not.

Note that full information on the approval of the study protocol must also be provided in the manuscript.

Animals and other research organisms

Policy information about studies involving animals; ARRIVE guidelines recommended for reporting animal research, and Sex and Gender in Research

Laboratory animals	6- to 8-week old Balb/c and Hsd:Athymic Nude Fox1nu Female mice. All animals were housed in the Transgenic Mouse Facility at Baylor College of Medicine (BCM) with a 12-hour day/12-hour night cycle in climate-controlled conditions.
Wild animals	No wild animals were used in the study.
Reporting on sex	This study exclusively used female mice to model TNBC in women only.
Field-collected samples	No field collected samples were used in the study.
Ethics oversight	The procedures used for in vivo animal models were conducted following protocol AN-504 approved by the Baylor College of Medicine Institutional Animal Care and Use Committee (IACUC).

Note that full information on the approval of the study protocol must also be provided in the manuscript.

Plants

Seed stocks	*Report on the source of all seed stocks or other plant material used. If applicable, state the seed stock centre and catalogue number. If plant specimens were collected from the field, describe the collection location, date and sampling procedures.*
Novel plant genotypes	*Describe the methods by which all novel plant genotypes were produced. This includes those generated by transgenic approaches, gene editing, chemical/radiation-based mutagenesis and hybridization. For transgenic lines, describe the transformation method, the number of independent lines analyzed and the generation upon which experiments were performed. For gene-edited lines, describe the editor used, the endogenous sequence targeted for editing, the targeting guide RNA sequence (if applicable) and how the editor was applied.*
Authentication	*Describe any authentication procedures for each seed stock used or novel genotype generated. Describe any experiments used to assess the effect of a mutation and, where applicable, how potential secondary effects (e.g. second site T-DNA insertions, mosiacism, off-target gene editing) were examined.*

Flow Cytometry

Plots

Confirm that:

☒ The axis labels state the marker and fluorochrome used (e.g. CD4-FITC).

☒ The axis scales are clearly visible. Include numbers along axes only for bottom left plot of group (a 'group' is an analysis of identical markers).

☐ All plots are contour plots with outliers or pseudocolor plots.

☐ A numerical value for number of cells or percentage (with statistics) is provided.

Methodology

Sample preparation	Cells were trypsinized to single cell suspension and labeled with reporters for flow cytometry.
Instrument	Attune NXT
Software	FlowJo
Cell population abundance	No cell sorting was involved.
Gating strategy	Single cells were gated based on FSC-A vs FSC-H. All single cells were used for analyses.

☐ Tick this box to confirm that a figure exemplifying the gating strategy is provided in the Supplementary Information.
